# Improving Reporting of Clinical Studies Using the POSEIDON Criteria: POSORT Guidelines

**DOI:** 10.3389/fendo.2021.587051

**Published:** 2021-03-19

**Authors:** Sandro C. Esteves, Alessandro Conforti, Sesh K. Sunkara, Luigi Carbone, Silvia Picarelli, Alberto Vaiarelli, Danilo Cimadomo, Laura Rienzi, Filippo Maria Ubaldi, Fulvio Zullo, Claus Yding Andersen, Raoul Orvieto, Peter Humaidan, Carlo Alviggi

**Affiliations:** ^1^ANDROFERT, Andrology and Human Reproduction Clinic, Campinas, Brazil; ^2^Department of Surgery (Division of Urology), University of Campinas (UNICAMP), Campinas, Brazil; ^3^Faculty of Health, Aarhus University, Aarhus, Denmark; ^4^Department of Neuroscience, Reproductive Science and Odontostomatology, University of Naples, Federico II, Naples, Italy; ^5^Department of Women’s Health, Faculty of Life Sciences, King’s College London, London, United Kingdom; ^6^Center for Reproductive Medicine, GENERA, Rome, Italy; ^7^Laboratory of Reproductive Biology, Faculty of Health and Medical Sciences, University Hospital of Copenhagen, Copenhagen, Denmark; ^8^Department of Obstetrics and Gynecology, Chaim Sheba Medical Center, Ramat Gan, Israel; ^9^Fertility Clinic Skive, Skive Regional Hospital, Skive, Denmark

**Keywords:** Patient-Oriented Strategies Encompassing IndividualizeD Oocyte Number (POSEIDON) criteria, ovarian stimulation, low prognosis, poor response, infertility, assisted reproductive technology, ART calculator, guidelines

## Abstract

The POSEIDON (**P**atient-**O**riented **S**trategies **E**ncompassing **I**ndividualize**D O**ocyte **N**umber) criteria were developed to help clinicians identify and classify low-prognosis patients undergoing assisted reproductive technology (ART) and provide guidance for possible therapeutic strategies to overcome infertility. Since its introduction, the number of published studies using the POSEIDON criteria has increased steadily. However, a critical analysis of existing evidence indicates inconsistent and incomplete reporting of critical outcomes. Therefore, we developed guidelines to help researchers improve the quality of reporting in studies applying the POSEIDON criteria. We also discuss the advantages of using the POSEIDON criteria in ART clinical studies and elaborate on possible study designs and critical endpoints. Our ultimate goal is to advance the knowledge concerning the clinical use of the POSEIDON criteria to patients, clinicians, and the infertility community.

## Introduction

The POSEIDON (**P**atient-**O**riented **S**trategies **E**ncompassing **I**ndividualize**D O**ocyte **N**umber) criteria were developed to identify and classify the low-prognosis patient undergoing assisted reproductive technology (ART) treatments (1–3). The new criteria’ primary objectives were to help clinicians delineate subtle differences in patients’ characteristics and provide guidance for possible stimulation strategies for these challenging patients classified as low prognosis (4, 5).

Women with low prognosis undergoing ART have defied clinicians for a long time, as no clear treatment strategies exist to improve outcomes significantly (6, 7). These women are characterized by a reduced chance of live birth after ART for at least two main issues: 1) reduced number of oocytes and, consequently, embryos; and 2) poor oocyte/embryo quality resulting from advanced female reproductive age (8–11).

**Figure 1:**
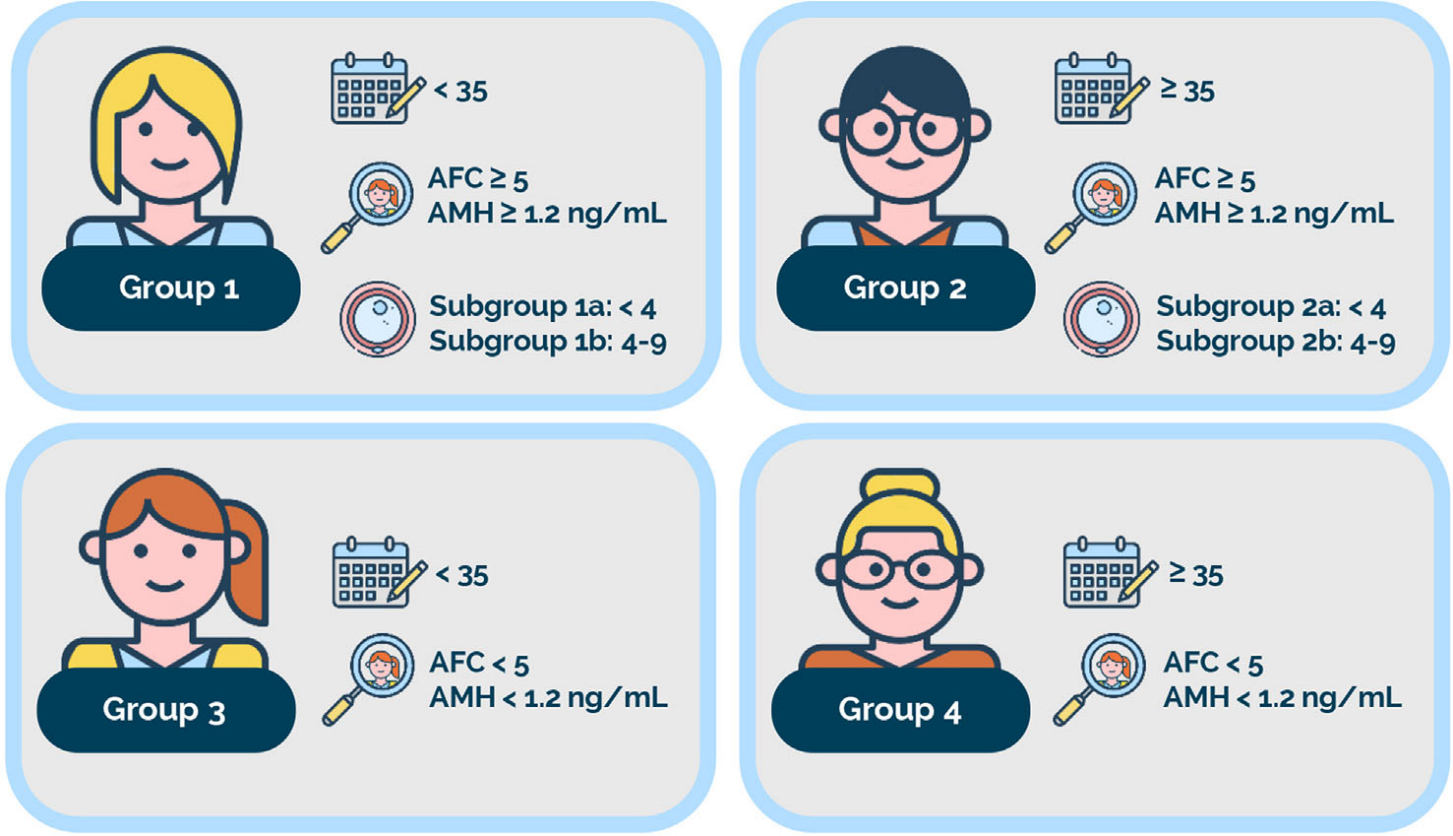
The POSEIDON criteria. Four distinct groups of low-prognosis patients can be established based on quantitative and qualitative parameters, namely: 1. The age of the patient and its related embryo aneuploidy rate; 2. Ovarian biomarkers [antral follicle count (AFC) and/or anti-Müllerian hormone (AMH)], and 3. The ovarian response in terms of oocyte quantity (if a previous cycle of conventional ovarian stimulation was carried out). Group 1: Patients <35 years with sufficient prestimulation ovarian reserve parameters (AFC ≥ 5, AMH ≥1.2 ng/ml) and with an unexpected poor (<4 oocytes) or suboptimal (four to nine oocytes) ovarian response. This group is further divided into subgroup 1a, constituted by patients with fewer than four oocytes; and subgroup 1b, constituted by patients with four to nine oocytes retrieved after standard ovarian stimulation, who, at any age, have a lower live birth rate than age-matched normal responders. Group 2: Patients ≥35 years with sufficient prestimulation ovarian reserve parameters (AFC ≥ 5, AMH ≥ 1.2 ng/ml) and with an unexpected poor or suboptimal ovarian response. This group is further divided into subgroup 2a, constituted by patients with fewer than four oocytes; and subgroup 2b, constituted by patients with four to nine oocytes retrieved after standard ovarian stimulation, who, at any age, have a lower live birth rate than age matched normal responders. Group 3: Patients <35 years with poor ovarian reserve prestimulation parameters (AFC < 5, AMH < 1.2 ng/ml). Group 4: Patients ≥35 years with poor ovarian reserve prestimulation parameters (AFC < 5, AMH < 1.2 ng/ml). Art drawing courtesy of Chloé Xilinas, Med.E.A., Rome, Italy.

Based on female age, ovarian biomarkers, and the number of oocytes retrieved, the low-prognosis patient is identified and further classified into four POSEIDON groups (**Figure 1**) (1, 4). Outside POSEIDON, patients without a low prognosis can be categorized based on their expected response to ovarian stimulation and hence their prognosis as a non-POSEIDON group. An important outcome that would set the POSEIDON and non-POSEIDON groups apart is the cumulative delivery rate (CDR) (4). In 2017, the International Committee for Monitoring Assisted Reproductive Technology (ICMART) defined the term as *‘the number of deliveries with at least one live birth resulting from one initiated or aspirated ART cycle, including all cycles in which fresh and/or frozen embryos are transferred, until one delivery with a live birth occurs or until all embryos are used, whichever occurs first, expressed per 100 cycles (initiated or aspirated)’* (12). On this basis, POSEIDON patients are expected to have lower CDR than non-POSEIDON patients overall. Moreover, CDRs are likely to differ across the four low-prognosis POSEIDON groups (4, 13, 14).

A critical backbone of the POSEIDON criteria is the number of oocytes retrieved –or expected to be retrieved– after a conventional ovarian stimulation with exogenous gonadotropins (1, 2, 4). The importance of oocyte numbers relates to its strong and independent association with the CDR (9, 11). Given that each oocyte has pregnancy potential, increased oocyte numbers may logically lead to higher CDR (8). The reason stems from the overall positive correlation between the numbers of oocytes retrieved and the resulting embryos obtained in *in vitro* fertilization/intracytoplasmic sperm injection (IVF/ICSI) treatment (15). Thus, the higher the embryo cohort, the higher the CDR, as more opportunities are available to achieve a pregnancy after transferring fresh and cryopreserved embryos (13, 14).

Ovarian markers, particularly antral follicle count (AFC) and anti-Müllerian hormone (AMH) levels, constitute another backbone of the POSEIDON criteria (16–19). These markers have been widely used in routine clinical practice to estimate ovarian response to gonadotropin stimulation in women undergoing ART. Despite their acceptability and overall good accuracy to predict poor and high ovarian responses, they cannot correctly uncover the so-called hypo-responder patient, who, despite having normal ovarian reserve markers like AFC and AMH, finish with an unexpected suboptimal low oocyte yield after conventional ovarian stimulation (20–22). These patients are included in the POSEIDON criteria (Groups 1 and 2), as the hypo-response decreases the number of oocytes retrieved, consequently impacting the CDRs (9).

To assess the ovarian response to stimulation, the POSEIDON group developed the ‘Follicle to-Oocyte Index’ (FOI). This index calculates the ratio between the total number of oocytes retrieved following conventional ovarian stimulation and the number of antral follicles at the start of stimulation (20). This new parameter better reflects the dynamic nature of follicular recruitment and might be adopted to assess the response to gonadotropin stimulation in all patients undergoing ART. This parameter is particularly informative to identify the patient with a suboptimal response to exogenous gonadotropin stimulation, typically observed in hypo-responders who usually have low FOIs. Accordingly, treatments aimed at increasing the FOI can be tested in interventional trials.

Lastly, female age, which has consistently shown to be the most predictive parameter for live birth in ART, is also included in the POSEIDON criteria. In ART, the older the woman, the lower the chances of reproductive success (23, 24). Thus, female age can be regarded as a proxy for oocyte/embryo genetic competence, given the well-established association between age and oocyte/embryo ploidy status (25, 26). Female age in the POSEIDON criteria is used to stratify the low-prognosis patients accordingly (**Figure 1**).

The decline in reproductive success is mainly attributed to higher oocyte aneuploidy rates in older women. However, the availability of euploid embryos for transfer increases the chances of having a baby, as sustained implantation rates after transfer of euploid embryos are about 50% and relatively independent of maternal age (27, 28). While blastocyst morphology and development speed (i.e., day of trophectoderm biopsy) do seem to impact the implantation potential of euploid embryos, and thus LBdR, maternal age has no apparent influence (29). In practical terms, the current evidence indicates that older women’s euploid embryos have similar implantation, live birth, and miscarriage rates than those of younger counterparts.

Accordingly, the POSEIDON group introduced a metric of success in ART, namely, the ability to retrieve the number of oocytes needed to obtain at least one euploid blastocyst for transfer in the specific patient (1, 2, 4). This number can be estimated without preimplantation genetic testing for aneuploidy (PGT-A), as embryo euploidy rates per age strata are well established (10, 25). The estimation can be performed manually using data from the literature or a dataset from an individual clinic or automatically using predictive models (30). According to the estimation, patient-oriented strategies can be elaborated to achieve the number of oocytes needed to obtain one euploid embryo and potentially increase success prospects (31–35).

## The Need to Improve The Quality of Clinical Studies Using The Poseidon Criteria

After introducing the POSEIDON criteria in 2015, several studies have explored its potential benefit in clinical practice (14, 34–46). However, a discrepancy has been noticed concerning the reporting of critical outcomes (36, 39, 40, 44–46). Failure to recognize the critical pillars of the POSEIDON criteria, as mentioned above, might limit the clinical utility of such studies, notably when the essential endpoints are incompletely reported or not reported at all (4, 13).

Studies looking at live birth rates in fresh cycles have shown that increased oocyte numbers are associated with increased live birth rates (8). However, reporting reproductive endpoints like clinical pregnancy, ongoing pregnancy, and even live birth may not necessarily reflect the impact of an enlarged oocyte or embryo cohort as a way to potentially increase the probability of pregnancy, particularly in the low prognosis patient (9, 11). Logically, having more embryos to transfer potentially increases the CDR. Along these lines, comparing two ovarian stimulation regimens that result in a similar number of oocytes retrieved might still reveal that one protocol is more efficient than the other for a specific low-prognosis patient group if an endpoint like the FOI was included in the study design. Lastly, a given ovarian stimulation strategy might result in more patients achieving the estimated oocyte number required to obtain at least one euploid embryo for transfer, thus indicating a better efficacy, which will only be recognized if this particular endpoint is included in the study design and analyzed accordingly.

Given the low-prognosis patient’s particularities and the steady increase in infertility studies using the POSEIDON criteria, we feel a need to clarify what to report and how to report. Therefore, to improve the quality of studies using the POSEIDON criteria, we developed a guideline based on the best evidence and expert judgment.

## Method

### Guideline Development

We developed the current guideline on behalf of the POSEIDON group (www.groupposeidon.com.br). The coordinators (SCE, CA, AC) assembled a guideline development group (GDG) composed of clinicians and researchers with experience developing and/or participating in infertility clinical trials. The group included both POSEIDON group members and non-members. It also included the editors-in-chief of two leading journals in reproductive medicine, ‘Frontiers in Endocrinology (Reproduction)’ and ‘Reproductive Biology and Endocrinology’.

The participants were given access to the relevant literature concerning the POSEIDON criteria and their related content. For this, a literature search was performed in PUBMED/MEDLINE from inception up to 20th July 2020, based on defined keywords (‘POSEIDON’, ‘Low-prognosis’, ‘Assisted Reproductive Technology’). A total of 41 articles were retrieved, including 11 review articles, 13 retrospective cohort studies, nine opinion/commentary/editorial articles, three articles concerning development and validation of predictive models, two prospective cohort studies, two letters to the editor, and one randomized controlled trial (RCT) (see **Supplementary Table 1** for a summary of published literature). The vast majority of articles were published in Frontiers in Endocrinology (23 articles), followed by Reproductive Biology and Endocrinology (three articles), Human Reproduction (three articles), and PLoS One (two articles) (1–5, 13, 14, 19, 20, 25, 30–60). The intention was to provide participants with the POSEIDON criteria’ conceptual features and the existing evidence on its clinical use.

The coordinators used the published CONSORT (Consolidated Standards of Reporting Trials), IMPRINT (Improving the Reporting of Clinical Trials of Infertility Treatments), STROBE (Strengthening the Reporting of Observational Studies in Epidemiology), and GRACE (Good Research for Comparative Effectiveness) statements as guidance to elaborate a list of items –with definitions– relevant to POSEIDON trials (61–64). The new statement was named **POSORT** (**PO**SEIDON **S**tatement **O**f **R**eporting **T**rials**)** guidelines. The document was circulated among participants, and a consensus was achieved on items to be reported and how. The group also achieved a consensus concerning the endpoints to be included in POSEIDON trials.

## Results

### POSEIDON Statement Of Reporting Trials (POSORT) Guidelines

The POSORT guidelines incorporate items on relevant quality dimensions of infertility care, including effectiveness, safety, and patient-centeredness (**Table 1**), which served as the basis for a 20-item checklist to be used by investigators in infertility trials using the POSEIDON criteria (**Supplementary Table 2**).

**Table 1:** Information to include when reporting studies using the POSEIDON criteria*.

**Title and abstract**	Identification as an observational study or randomized trial using the POSEIDON criteria.
**Introduction**	Explanation of rationale, specific objectives or hypotheses, and how the study may help to advance knowledge concerning the POSEIDON concept.
**Methods**	
*Participants*	Inclusion and exclusion criteria must be clearly defined; Characterize how infertility factors in participants were evaluated, describe the definitions used, and the settings where the data were collected; Define which ovarian marker, AFC or AMH or both, was used to classify the patients as per the POSEIDON criteria, and describe the methods for AFC/AMH measurements; In POSEIDON groups 1 and 2 studies, previous ovarian stimulation should be characterized; The preferred unit of analysis is ‘patient’ rather than ‘cycle’.
*Interventions*	Characterize the intervention (if applicable) and state the duration of the intervention noting when the treatment started and concluded. State the temporal relation of the intervention to pregnancy.
*Outcomes*	Clearly define the primary outcome. When more than one embryo transfer cycle occurs, the preferred outcome is cumulative delivery rate per initiated or aspiration cycle; Both male and female outcomes, other than cumulative delivery rate, could be the primary outcome and should be justified. However, when cumulative delivery rate is not the primary endpoint and embryos are transferred, reproductive outcomes (e.g., live birth rate, ongoing pregnancy rate, miscarriage rate, time to delivery rate) should be reported; Efforts should be made to include live birth data, including gestational age, birthweight, and sex of infant; Clearly define predictors, potential confounders, and effect modifiers. Describe how confounders were adjusted for.
*Data collection and analysis*	In observational studies, particularly the ones using real-world data, explain features of electronic medical records utilized, including how data quality was verified (e.g., completeness of data, availability of data on exposure, outcomes, and covariates); Describe statistical methods, including those used to control for confounders, sensitivity analyses, and how the sample size was determined.
**Results**	State the duration of infertility (including whether it is primary or secondary), relevant infertility treatment history, and cause of infertility in women and men. Report the numbers of couples/patients who were screened and eligible, and describe (in observational studies) the proportion of patients fitting each POSEIDON group and those classified as non-POSEIDON; Report numbers of individuals completing the follow-up and analyzed, and consider the use of a flow diagram; Provide unadjusted and confounder-adjusted estimates with precision (e.g., 95% confidence interval), and other analyses carried out (e.g., subgroup and sensitivity analyses) Report harms^¶^ or unintended effects in each group (men, women, infants) during treatment (including both male and female partners), during pregnancy, and around birth, and in infants after birth.
**Discussion**	Discuss generalizability of the study findings and how the results compare to other studies using the POSEIDON concept; Discuss trial limitations, including, but not limited to potential bias and imprecision (factors & interventions affecting endpoints should be discussed as ‘associations’ rather than ‘causation’ in observational studies).

*We recommend application of these guidelines in conjunction with the CONSORT, IMPRINT, STROBE, and GRADE guidelines as appropriate (see http://www.consort-statement.org/; https://strobe-statement.org/; https://www.graceprinciples.org/).

^¶^Reportable harms include OHSS, infection, bleeding, multiple pregnancy and maternal pregnancy complications, and harms or unintended effects on the fetus/newborn, including congenital abnormalities, and major neonatal complications as well as infant developmental delays or medical problems.

AFC, antral follicle count; AMH, anti-Müllerian hormone.

A list of endpoints is provided in **Table 2**. The GDG considered that CDR, as defined by ICMART (12), should be the preferred primary endpoint in intervention trials using the POSEIDON criteria. The recommended secondary endpoints include the number of oocytes retrieved (both overall and metaphase II oocytes), the number of embryos generated, the FOI (20), and how effective a specific intervention was in achieving the number of oocytes estimated by the ART calculator (30). Time to live birth (TTLB) is an additional outcome that should be considered, given that a shorter time in achieving a live birth is a reflection of the clinical and cost-effectiveness of any intervention (65). Also, in observational studies, particularly those involving big analytics, the frequency of patients fitting each POSEIDON group should be reported, including –if possible– a control group of non-POSEIDON patients for comparison. Other endpoints can be included but must be justified. A list of additional endpoints that may merit reporting is provided in **Table 3**.

**Table 2:** POSEIDON endpoints.

Endpoint	Definition
Cumulative delivery rate (CDR)*	Number of deliveries with at least one live birth resulting from one initiated, aspirated, or embryo transfer ART cycle, including all cycles in which fresh and/or frozen embryos are transferred, until one delivery with a live birth occurs or until all embryos are used, whichever occurs first, expressed per 100 cycles (the denominator must be specified. i.e., initiated or aspirated cycles)
Time to pregnancy/Time to live birth (TTP/TTLB)	The time taken to establish a clinical pregnancy or live birth, measured in days or in number of treatment cycles
Follicle-to-oocyte index (FOI)	Ratio between the number of oocytes retrieved at oocyte pick-up and the number of antral follicles (AFC) at the start of stimulation
Number of oocytes retrieved	Total number of oocytes retrieved after oocyte pick-up
Number of metaphase II oocytes	Total number of metaphase II oocytes retrieved after oocyte pick-up
Number of embryos generated	Total number of viable embryos^‡^ generated after an IVF or ICSI cycle
Percentage of patients who achieved the minimum number of metaphase II oocytes estimated by the ART calculator	The ART calculator is a clinical predictive model that estimates, prior to treatment, the minimum number of metaphase II oocytes (MIImin) (and the 95% confidence interval of that number) needed to obtain at least one euploid blastocyst^¶^
Prevalence of low prognosis (POSEIDON) and non-low prognosis (Non-POSEIDON)	Frequency (%) of POSEIDON patients (by subgroup) and non-POSEIDON patients in the cohort^§^

*Live birth: any delivery of a live infant ≥22 weeks’ gestation (fetus exiting the body with signs of life: movement, breathing, heartbeat).

^‡^The embryo stage must be specified (cleavage, blastocyst).

^¶^The probability of success (e.g., 70%, 80%, and 90%) used for the estimation should be specified.

^§^Observational studies, including real-world data analysis.

AFC, antral follicle count; ART, assisted reproductive technology; IVF, in vitro fertilization; ICSI, intracytoplasmic sperm injection.

**Table 3:** Other endpoints that merit reporting.

Endpoint	Definition and formula
Live birth* delivery rate (LBdR)	Number of deliveries that resulted in at least one live birth, expressed per 100 cycle attempts (initiated, aspirated, transfer cycles).
Ongoing Pregnancy rate (OPR)	Number of viable intrauterine pregnancies of at least 12 weeks duration confirmed on ultrasound scan per 100 clinical pregnancies
Time-to-live birth	The time taken to achieve a live birth, measured in days or in number of treatment cycles, start time point from oocyte retrieval and end time point the day of delivery.
Multiple birth rate	Number of multiple births, defined by the complete expulsion or extraction of ≥1 fetus, after ≥ 22 wks. gestational age (e.g., twin delivery = two births) per 100 deliveries
Miscarriage rates	Number of spontaneous losses of clinical pregnancies before 22 completed weeks of gestational age per 100 clinical pregnancies

*Live birth, any delivery of a live infant ≥22 weeks’ gestation (fetus exiting the body with signs of life: movement, breathing, heartbeat).

The justifications and discussion on the key elements of the POSORT guidelines are provided in the next sections.

## Discussion

### Advantages of POSEIDON Criteria in Clinical ART Trials

The likelihood of delivering a live born decreases progressively with female age (23, 24). Although this effect may be partially modulated by ovarian reserve, paternal factors, and the number of oocytes and embryos obtained after ovarian stimulation, the impaired reproductive outcome in the aging woman is primarily related to the increased oocyte/embryo aneuploidy rate (8–11, 13, 25, 26, 51). Indeed, the probability of having euploid embryos decreases progressively with age, being ≥50% and <50% overall, when a threshold of 35 years is used (25). Despite this given fact, an increased oocyte yield might lead to more embryos available for transfer, which would provide the patient with a better prospect when the transfer of fresh and frozen-thawed embryos is considered. Indeed, existing data indicate that the number of oocytes is strongly and independently associated with CDR (11, 14).

POSEIDON patients have an overall lower CDR than non-POSEIDON patients (13, 14, 42). However, the prognosis varies according to subgroup. In a recent large retrospective analysis involving 18,455 cycles, the authors showed a progressive decrease in CDR across POSEIDON groups (42). In this study, the CDR was 44.6% in Group 1, 35.5% in Group 3, 24.5% in Group 2, and 12.7% in Group 4. Notably, a significantly higher CDR was observed in women who did not fulfill the POSEIDON criteria (non-POSEIDON) than those who did. These findings are consistent with a recent Dutch multicenter observational cohort study in which differences in pregnancy rates among POSEIDON groups were also observed (40). In both studies, the female age emerged as impacting the reproductive prognosis more than the ovarian reserve and the number of oocytes retrieved. Nonetheless, these and other studies (13, 14) indicate that CDR in the POSEIDON patient is affected not only by oocyte/embryo quality (i.e., female age) but also by oocyte quantity.

The existing evidence, albeit limited, collectively suggest that the POSEIDON criteria are overall useful to prognosticate reproductive outcomes among women undergoing ART, in which each group might demand specific treatment strategies (4, 5, 21, 31–35, 38, 39, 45, 54–57, 60). Thus, besides providing a counseling tool, the POSEIDON criteria may guide clinical management to optimize the FOI. Improving oocyte yield with a consequent higher number of embryos may result in a higher chance of having a euploid embryo transferred (10, 25). Transfer of a euploid embryo potentially results in an increased implantation rate and shortened TTLB. Given each POSEIDON subgroup is characterized by a more homogenous population with specific prognostic characteristics, we encourage clinicians to move from the existing heterogeneous definitions of poor responders to the low-prognosis notion proposed by the POSEIDON group.

### Biomarkers’ Considerations

The POSEIDON criteria are simple and straightforward as regards thresholds to defining each subgroup. For instance, unlike other criteria that apply an ill-defined ovarian reserve threshold (66), the POSEIDON classification uses objective thresholds of antral follicle count (AFC) and Anti-Müllerian hormone (AMH) values. According to the POSEIDON stratification, a rigorous and precise assessment of AFC and/or AMH is necessary before starting a clinical trial. For an adequate AFC evaluation, the criteria proposed by Broekmans and co-workers in 2010 (16) and Coelho Neto and co-workers in 2017 (67) seem appropriate. These practical guidelines summarize the main technical aspects for performing AFC, including the optimal machine settings, time of menstrual cycle (e.g., early follicular phase, which follicles to measure and how, and clinical considerations. However, inter-observer and intra-observer variability in AFC determination has been reported (68), and the use of two-dimensional transvaginal sonography may yield different results even by experienced operators (69). The adoption of automated ultrasonographic assessments could also be considered (70). Manual and automated methods did not differ in terms of fertility outcome (71); however, the automatized method seems to offer a lower intra- and inter-observed variability than standard 2D methods (70).

Along these lines, several enzyme-linked immunosorbent assays (ELISA) have been developed for AMH assessments (72), and manual assays were recently replaced by fully automatized assays (73, 74). Despite this, the reliability of some assays has been questioned due to technical issues, and it has been suggested that the existing automated methods cannot be used interchangeably as their results do not necessarily line up (75). For example, automated assays generate lower values than ELISA, and POSEIDON thresholds were based on Gen II ELISA. Therefore, POSEIDON AMH thresholds must be converted if an automated assay (e.g., Elecsys) is utilized (76). Nonetheless, a recent multicenter study showed that the area under the curve (AUC) for predicting poor response, using an AMH automated assay, was 0.929, compared to previous data of 0.78 (73).

Clinicians relying on AMH to assess ovarian reserve must understand the existing assays’ technical limitations. The AMH assay used should be standardized, and if possible, calibrated against other assays. At this point, however, it might be advisable to use a single assay in the clinic with precise thresholds to distinguish between patients expected to have a poor, normal, or high response to ovarian stimulation. Apart from this, factors potentially affecting AMH results should be considered, including oral contraceptives used for cycle synchronization before OS in GnRH antagonist regimens (17, 77).

Collectively, the POSEIDON criteria underline the importance of correctly classifying infertility patients undergoing ART. The classification system emphasizes the impact of female age and its related oocyte and embryo’s aneuploidy rates, and the number of oocytes retrieved for ART success. It also underlines that treatment delays should be avoided in the low-prognosis infertility patient.

### Study Design Considerations

Rigorous planning and strict execution are critical parameters in performing high-quality studies. The time invested in planning usually pays off in the end. Having acknowledged the heterogeneity of the low prognosis group of patients undergoing ART, researchers need to focus on well-defined subgroups to test specific interventions. In this regard, the POSEIDON criteria are advantageous in terms of providing a more homogeneous patient grouping.

Among different study designs, it is widely recognized that RCTs represent the optimal way to verify the clinical efficacy of specific interventions (59, 60). In RCTs, participants are prospectively and randomly allocated to either intervention or another, following strict inclusion and exclusion criteria. The CONSORT and IMPRINT statements have provided useful guidance to increase the quality of infertility trials (61, 62). These guidelines also served as the basis for the development of the current POSORT guidelines.

Although RCTs remain the backbone of high-quality evidence (78), an overwhelming majority of infertility patients are treated outside the scope of such studies. Besides, most patients treated in routine clinical practice do not necessarily meet the inclusion and exclusion criteria adopted in RCTs (79). Importantly, valuable information can be obtained from data generated during routine clinical practice using pragmatic clinical trials and observational studies (63, 64). These study designs may provide valid information on how therapy affects a heterogeneous infertility population (e.g., those who are older and those with concomitant medical problems, impaired ovarian function, and diverse ethnicities/races). Observational studies can also generate hypotheses for testing in RCTs, assess trial practicability by assessing the impact of planned inclusion/exclusion criteria in the pertinent population, informing about probability distributions to be used in statistical analyses, and identifying prognostic factors or patient baseline attributes for improvement or stratification.

If well conducted, observational studies may generate real-world evidence, which refers to evidence generated from clinically relevant data gathered outside of the conditions imposed by conventional RCTs (78, 80). These data can be collected from various sources, including registries (at a country or region level) and electronic health records (at a site level). Such studies are less time-consuming and less expensive than RCTs and allow individual fertility centers to contribute their specific experience on how treatments work in real-world settings. However, minimum standards should be followed to secure the quality of observational studies. Given treatment decisions might be driven by many factors (performance bias), and real-world patients can have complex clinical conditions (selection bias), studies must address unbalanced groups, confounders, differential follow-ups, and missing data (64). Thus, our guidelines have also taken into consideration the STROBE and GRACE recommendations.

### Endpoints in POSEIDON Criteria Clinical Studies

Several preclinical (e.g., cumulative gonadotropin dose, number of oocytes retrieved, metaphase II oocyte rate, 2PN rate, blastulation rate; post-ICSI degeneration rate, survival rates (embryo/oocyte/sperm) post-warming) and clinical endpoints (e.g., clinical pregnancy rate, ongoing pregnancy rate, live birth delivery rate [LBdR], CDR, TTLB, multiple birth rate, OHSS rates) are used in ART clinical trials. As mentioned above, the number of oocytes retrieved is strictly related to live births. Thus, this parameter represents an important surrogate endpoint that should be pursued in clinical trials devoted to POSEIDON patients. Moreover, the POSEIDON group proposed an innovative method to assess ovarian sensitivity by introducing the FOI, which measures the efficiency of the ovarian stimulation protocol and the ovarian resistance to gonadotropin stimulation. The FOI is defined by the ratio between the number of oocytes retrieved at the end of the ovarian stimulation in relation to the AFC at the beginning of stimulation (20). The FOI may be informative, especially in patients with unexpected suboptimal or poor responses to ovarian stimulation (i.e., POSEIDON groups 1 and 2). In these patients, the primary aim of interventional trials would be to identify strategies to overcome suboptimal response to ovarian stimulation, like personalizing FSH starting dosage based on specific genotype characteristics, supplementing with recombinant luteinizing hormone, or modifying the trigger strategy (21, 22, 31, 34, 47, 54, 60). On this basis, the FOI may serve as a marker to identify patients with a relative FSH/LH deficiency who could benefit from individualized ovarian stimulation.

As for reproductive endpoints, the LBdR –defined as the number of deliveries that resulted in at least one live birth obtained after 22 weeks’ gestation, expressed per 100 cycle attempts (initiated, aspirated, or embryo transfer cycles)–, and more recently, the CDR represent essential endpoints for patients, clinicians, and the public when evaluating the effects of treatment (12). Among these, the CDR following the transfer of fresh and/or frozen embryos obtained from a single initiated/aspiration cycle represents the best way to evaluate ART success in POSEIDON studies.

We recognize that the CDR might be difficult to obtain because this implies that all useful embryos should be transferred and allowed to have a chance to develop into a live born. Indeed, some patients will end up not using all their embryos, and if they do, it will often take a considerable period to complete the trial. However, the number of oocytes and embryos in POSEIDON patients is overall low, thus allowing an account of the outcome of all embryos and therefore generating a true CDR.

Live birth endpoints could also be challenging in low responders and advanced age patients, given the noticed age-dependent miscarriage rate (23). A significant treatment discontinuation rate before delivery may also be noted during trials, making the sample size required to analyze such endpoints less practical. For instance, intrauterine fetal death is observed in about 5% of ongoing IVF pregnancies after a 12-week gestation period, a risk that is further increased in older women (81). Consequently, large RCTs have opted to use other primary endpoints than live births (81). Clinical endpoints such as implantation rates, ongoing pregnancy rates, and miscarriage rates are also clinically significant as they represent intermediate outcomes reflecting the continuum of the ART process (82, 83). However, the use of such endpoints in preference over CDR in POSEIDON studies should always be justified. When considering time-to-pregnancy (TTP) or TTLB as an outcome, the start time point should be oocyte retrieval and the end time point the clinical pregnancy (TTP) or live birth (TTLB) (65). As POSEIDON interventions should aim to increase the oocyte yield for the low prognosis patients, this justifies the start point from oocyte retrieval for TTP and TTLB outcomes. Lastly, we recommend a more comprehensive reporting of outcomes in POSEIDON trials, including potential harms and health of the resulting offspring (**Table 1**).

### The ART Calculator

To establish a valuable working plan for low prognosis patients and improve patient counseling, the POSEIDON group, as previously mentioned, proposed a novel metric of success in ART, namely, the retrieval of a sufficient number of oocytes to achieve at least one euploid embryo for transfer (1, 2, 4). In this context, Esteves and co-workers, on behalf of the POSEIDON group, developed a predictive model to determine the minimum number of oocytes required to obtain at least one blastocyst for transfer (30). In their study, female age, sperm source for IVF/ICSI (ejaculated vs. testicular sperm), and the number of oocytes retrieved were the main predictors affecting the blastocyst euploid probability. In practical terms, the predictive model estimates the optimal average number of metaphase II (MII) oocytes (and the 95% confidence interval), which increases progressively with aging and is magnified further by the use of testicular sperm from patients with nonobstructive azoospermia (30).

The ART calculator estimations may be adopted in POSEIDON clinical trials as a novel endpoint to determine the effectiveness of the interventions used. For example, the proportion of POSEIDON patients reaching the target number of MII oocytes as per the ART calculator could be determined and compared, and results analyzed in terms of how they translated to pregnancy success. Besides estimating the number of MII oocytes for at least one euploid blastocyst, the calculator also estimates the chance of having a euploid blastocyst based on the real number of oocytes retrieved. Thus, even if the ideal number of MII oocytes is not achieved, the probability of having a euploid blastocyst could be compared according to the interventions investigated. The latter might be of particular relevance to the advanced age POSEIDON patient, in whom the calculated ideal number of oocytes is more challenging to achieve.

The ART calculator was recently validated in a multicenter study (51). In detail, clinical and embryological data of 1,464 consecutive infertile couples subjected to IVF/ICSI and PGT-A were assessed. The authors demonstrated that the estimations provided by the ART calculator were strongly correlated with the actual probability of blastocyst euploidy per MII oocyte (r = 0.91) and the minimum number of MII oocytes to obtain at least one euploid blastocyst (r = 0.88).

In summary, besides being a new tool to be used both in clinical practice for counseling and treatment planning, the ART calculator could be a useful tool in POSEIDON clinical trials to compare treatments and strategies between study and control groups balancing both quantity (number of oocytes collected) and quality (euploidy of embryos).

### Strengths and Limitations

The POSORT guidelines have several strengths. They were developed by an international panel of experts in reproductive medicine, many of which are members of the POSEIDON group. The group reached a consensus on the minimum standards for relevant clinical studies using the POSEIDON criteria. The consensus was based mainly on a detailed and critical analysis of the available literature concerning the POSEIDON criteria.

However, our guidelines have some limitations. First, the number of published studies on POSEIDON criteria is still limited. Therefore, evidence from other relevant studies and expert experience were also considered, and the current version may not represent an exhaustive list of statements. Additionally, the guidelines only represent the opinion of the expert included. Along these lines, patient representatives were not included. Despite these limitations, the POSORT guidelines are the first of their kind to provide an expert opinion on specific approaches to be considered in POSEIDON studies. As with all guidelines, ours is an evolving document that should be revised periodically as new evidence emerges. The perspectives provided in this consensus complement existing guidelines and may help advance knowledge, potentially improving treatment outcomes.

## Conclusions

We developed guidelines to improve the quality of reporting in clinical infertility studies using the POSEIDON criteria. Our aims are to help researchers better characterize the study participants and report critical endpoints relevant to the POSEIDON framework. The ultimate goal is to promote complete and consistent reporting to advance knowledge concerning the POSEIDON criteria’s clinical utility.

## Author Contributions

SE and AC coordinated the GDG and had a leading role in collecting the evidence, drafting the manuscript, and handling the GDG’s comments. All participants contributed to the guideline development, evidence summary and recommendations, and writing sections of the manuscript. All authors contributed to the article and approved the submitted version.

## Conflict of Interest

SE and CA declare receipt of unrestricted research grants from Merck and lecture fees from Merck. SS declares the receipt of honorarium for lectures from Merck, MSD, and Ferring. PH has received unrestricted research grants from MSD, Merck, and Ferring as well as honoraria for lectures from MSD, Merck, Gedeon–Richter, Theramex, and IBSA. CYA has received unrestricted grants from Gedeon-Richter and honoraria for lectures from IBSA, Ferring, and Merck. FU and AV have received honoraria for lectures from MSD and Merck. The funders listed above had no involvement with the study.

The remaining authors declare that the research was conducted in the absence of any commercial or financial relationships that could be construed as a potential conflict of interest.
