# Supplementary material for: Improving Reporting of Clinical Studies Using the POSEIDON Criteria: POSORT Guidelines
Source: Front Endocrinol (Lausanne). 2021 Mar 19;12:587051. doi: 10.3389/fendo.2021.587051 (PMC8017440; doi:10.3389/fendo.2021.587051)
Supplement: Supplementary file 1 [file Table_1.docx]

**Supplementary Table 1. POSEIDON criteria summary of literature**

| **Study title** | **Authors** | **Year** | **Journal** | **Type** |
| --- | --- | --- | --- | --- |
| Dual-trigger improves the outcomes of in vitro fertilization cycles in older patients with diminished ovarian reserve: A retrospective cohort study. | Chern CU, Li JY, Tsui KH, Wang PH, Wen ZH, Lin LT. | 2020 | PLoS One. | Retrospective cohort study |
| Cumulative live-birth, perinatal and obstetric outcomes for POSEIDON groups after IVF/ICSI cycles: a single-center retrospective study. | Abdullah RK, Liu N, Zhao Y, Shuang Y, Shen Z, Zeng H, Wu J. | 2020 | Sci Rep. | Retrospective cohort study |
| Efficacies of different ovarian hyperstimulation protocols in poor ovarian responders classified by the POSEIDON criteria. | Li F, Ye T, Kong H, Li J, Hu L, Jin H, Su Y, Li G. | 2020 | Aging (Albany NY) | Retrospective cohort study |
| Live birth rates of low prognosis patients according to POSEIDON criteria; A retrospective cohort study. | Seven B, Gulerman C, Ozgu-Erdinc AS, Yilmaz N, Engin-Ustun Y. | 2020 | J Gynecol Obstet Hum Reprod. | Retrospective cohort study |
| COVID-19 and assisted reproductive technology services: repercussions for patients and proposal for individualized clinical management. | Alviggi C, Esteves SC, Orvieto R, Conforti A, La Marca A, Fischer R, Andersen CY, Bühler K, Sunkara SK, Polyzos NP, Strina I, Carbone L, Bento FC, Galliano D, Yarali H, Vuong LN, Grynberg M, Drakopoulos P, Xavier P, Llacer J, Neuspiller F, Horton M, Roque M, Papanikolaou E, Banker M, Dahan MH, Foong S, Tournaye H, Blockeel C, Vaiarelli A, Humaidan P, Ubaldi FM; POSEIDON (Patient-Oriented Strategies Encompassing IndividualizeD Oocyte Number) group | 2020 | Reprod Biol Endocrinol. | Opinion |
| Follicular Output Rate and Follicle-to-Oocyte Index of Low Prognosis Patients According to POSEIDON Criteria: A Retrospective Cohort Study of 32,128 Treatment Cycles. | Chen L, Wang H, Zhou H, Bai H, Wang T, Shi W, Shi J. | 2020 | Front Endocrinol. (Lausanne) | Retrospective cohort study |
| [Novel Physiology and Definition of Poor Ovarian Response; Clinical Recommendations.](https://pubmed.ncbi.nlm.nih.gov/32204404/) | Abu-Musa A, Haahr T, Humaidan P. | 2020 | Int J Mol Sci. | Review |
| Management Strategies for POSEIDON Group 2. | Sunkara SK, Ramaraju GA, Kamath MS. | 2020 | Front Endocrinol. (Lausanne) | Commentary |
| [Commentary: Management Strategies for POSEIDON Groups 3 and 4.](https://pubmed.ncbi.nlm.nih.gov/32117060/) | Fischer R, Baukloh V. | 2020 | Front Endocrinol. (Lausanne) | Commentary |
| Commentary: Management Strategies for POSEIDON Groups 3 and 4. | Bühler KF. | 2020 | Front Endocrinol.  (Lausanne) | Commentary |
| Validation of ART Calculator for Predicting the Number of Metaphase II Oocytes Required for Obtaining at Least One Euploid Blastocyst for Transfer in Couples Undergoing in vitro Fertilization/Intracytoplasmic Sperm Injection. | Esteves SC, Yarali H, Ubaldi FM, Carvalho JF, Bento FC, Vaiarelli A, Cimadomo D, Özbek İY, Polat M, Bozdag G, Rienzi L, Alviggi C. | 2020 | Front Endocrinol. (Lausanne) | Predictive model validation study |
| \| [The POSEIDON Criteria and Its Measure of Success Through the Eyes of Clinicians and Embryologists.](https://pubmed.ncbi.nlm.nih.gov/31824427/) \| \| --- \| | Esteves SC, Alviggi C, Humaidan P, Fischer R, Andersen CY, Conforti A, Bühler K, Sunkara SK, Polyzos NP, Galliano D, Grynberg M, Yarali H, Özbek IY, Roque M, Vuong LN, Banker M, Rienzi L, Vaiarelli A, Cimadomo D, Ubaldi FM. | 2019 | Front Endocrinol. (Lausanne) | Perspective |
| Dehydroepiandrosterone Supplementation Improves the Outcomes of in vitro Fertilization Cycles in Older Patients With Diminished Ovarian Reserve | Chen SN, Tsui KH, Wang PH, Chern CU, Wen ZH, Lin LT. | 2019 | Front Endocrinol. (Lausanne) | Retrospective cohort study |
| The Effect of Growth Hormone on the Clinical Outcomes of Poor Ovarian Reserve Patients Undergoing in vitro Fertilization/Intracytoplasmic Sperm Injection Treatment: A Retrospective Study Based on POSEIDON Criteria | Cai MH, Gao LZ, Liang XY, Fang C, Wu YQ, Yang X. | 2019 | Front Endocrinol. (Lausanne) | Retrospective cohort study |
| Oocyte quantity, as well as oocyte quality, plays a significant role for the cumulative live birth rate of a POSEIDON criteria patient. | Esteves SC, Roque M, Sunkara SK, Conforti A, Ubaldi FM, Humaidan P, Alviggi C. | 2019 | Hum Reprod. | Letter to the Editor |
| [Reply: The low responder according to the POSEIDON criteria: is the prognosis really poor?](https://pubmed.ncbi.nlm.nih.gov/31756245/) | Leijdekkers JA, Torrance HL, Broekmans FJM. | 2019 | Hum Reprod. | Letter to the Editor |
| Management Strategies for POSEIDON's Group 1. | Polyzos NP, Drakopoulos P. | 2019 | Front Endocrinol. (Lausanne) | Commentary |
| Cumulative Live Birth Rates in Low Prognosis Patients According to the POSEIDON Criteria: An Analysis of 26,697 Cycles of in vitro Fertilization/Intracytoplasmic Sperm Injection. | Li Y, Li X, Yang X, Cai S, Lu G, Lin G, Humaidan P, Gong F. | 2019 | Front Endocrinol. (Lausanne) | Retrospective cohort study; real-world evidence |
| Management Strategies for POSEIDON's Groups 3 and 4. | Haahr T, Dosouto C, Alviggi C, Esteves SC, Humaidan P. | 2019 | Front Endocrinol. (Lausanne) | Review |
| [Ovarian stimulation modalities in poor responders.](https://pubmed.ncbi.nlm.nih.gov/31385487/) | Özkan ZS. | 2019 | Turk J Med Sci. | Review |
| Androgens Profile in Blood Serum and Follicular Fluid of Women With Poor Ovarian Response During Controlled Ovarian Stimulation Reveals Differences Amongst POSEIDON Stratification Groups: A Pilot Study. | Fuentes A, Sequeira K, Tapia-Pizarro A, Muñoz A, Salinas A, Céspedes P, Escalona J, Godoy A. | 2019 | Front Endocrinol. (Lausanne) | Prospective cohort study |
| Future Perspectives of POSEIDON Stratification for Clinical Practice and Research | Humaidan P, La Marca A, Alviggi C, Esteves SC, Haahr T. | 2019 | Front Endocrinol. (Lausanne) | Review |
| [Management of Women With an Unexpected Low Ovarian Response to Gonadotropin.](https://pubmed.ncbi.nlm.nih.gov/31316461/) | Conforti A, Esteves SC, Cimadomo D, Vaiarelli A, Di Rella F, Ubaldi FM, Zullo F, De Placido G, Alviggi C | 2019 | Front Endocrinol. (Lausanne) | Review |
| Cumulative Live Birth Rates of Good and Low Prognosis Patients According to POSEIDON Criteria: A Single Center Analysis of 18,455 Treatment Cycles. | Shi W, Zhou H, Tian L, Zhao Z, Zhang W, Shi J. | 2019 | Front Endocrinol. (Lausanne) | Retrospective cohort study; real-world evidence |
| An Observational Retrospective Cohort Trial on 4,828 IVF Cycles Evaluating Different Low Prognosis Patients Following the POSEIDON Criteria | Levi-Setti PE, Zerbetto I, Baggiani A, Zannoni E, Sacchi L, Smeraldi A, Morenghi E, De Cesare R, Drovanti A, Santi D. | 2019 | Front Endocrinol. (Lausanne) | Retrospective cohort study; real-world evidence |
| Ovarian Reserve Markers to Identify Poor Responders in the Context of Poseidon Classification | Grisendi V, Mastellari E, La Marca A. | 2019 | Front Endocrinol. (Lausanne) | Review |
| Cumulative Live Birth Rates in Low-Prognosis Women. | Leijdekkers JA, Eijkemans MJC, van Tilborg TC, Oudshoorn SC, van Golde RJT, Hoek A, Lambalk CB, de Bruin JP, Fleischer K, Mochtar MH, Kuchenbecker WKH, Laven JSE, Mol BWJ, Torrance HL, Broekmans FJM; OPTIMIST study group. | 2019 | Hum Reprod. | Prospective cohort study |
| Understanding Follicular Output Rate (FORT) and Its Implications for POSEIDON Criteria | Grynberg M, Labrosse J. | 2019 | Front Endocrinol. (Lausanne) | Perspective |
| A Novel Predictive Model to Estimate the Number of Mature Oocytes Required for Obtaining at Least One Euploid Blastocyst for Transfer in Couples Undergoing in vitro Fertilization/Intracytoplasmic Sperm Injection: The ART Calculator. | Esteves SC, Carvalho JF, Bento FC, Santos J. | 2019 | Front Endocrinol. (Lausanne) | Predictive model development study |
| GnRH Agonist Long Protocol Versus GnRH Antagonist Protocol for Various Aged Patients With Diminished Ovarian Reserve: A Retrospective Study | Huang MC, Tzeng SL, Lee CI, Chen HH, Huang CC, Lee TH, Lee MS. | 2018 | PLoS One. | Retrospective cohort study |
| Understanding Ovarian Hypo-Response to Exogenous Gonadotropin in Ovarian Stimulation and Its New Proposed Marker-The Follicle-To-Oocyte (FOI) Index. | Alviggi C, Conforti A, Esteves SC, Vallone R, Venturella R, Staiano S, Castaldo E, Andersen CY, De Placido G. | 2018 | Front Endocrinol. (Lausanne) | Review |
| Defining Low Prognosis Patients Undergoing Assisted Reproductive Technology: POSEIDON Criteria-The Why. | Esteves SC, Roque M, Bedoschi GM, Conforti A, Humaidan P, Alviggi C. | 2018 | Front Endocrinol.  (Lausanne) | Review |
| Novel Approaches for Diagnosis and Management of Low Prognosis Patients in Assisted Reproductive Technology: The POSEIDON Concept | Conforti A, Esteves SC, Picarelli S, Iorio G, Rania E, Zullo F, De Placido G, Alviggi C. | 2018 | Panminerva Med. | Review |
| Double Stimulation in the Same Ovarian Cycle (DuoStim) to Maximize the Number of Oocytes Retrieved From Poor Prognosis Patients: A Multicenter Experience and SWOT Analysis. | Vaiarelli A, Cimadomo D, Trabucco E, Vallefuoco R, Buffo L, Dusi L, Fiorini F, Barnocchi N, Bulletti FM, Rienzi L, Ubaldi FM. | 2018 | Front Endocrinol. (Lausanne) | Retrospective cohort study |
| Estimation of Age-Dependent Decrease in Blastocyst Euploidy by Next Generation Sequencing: Development of a Novel Prediction Model. | Esteves SC, Carvalho JF, Martinhago CD, Melo AA, Bento FC, Humaidan P, Alviggi C; POSEIDON (Patient-Oriented Strategies Encompassing IndividualizeD Oocyte Number) Group | 2019 | Panminerva Med. | Retrospective cohort study and prediction model development |
| What is new in the management of poor ovarian response in IVF? | Vaiarelli A, Cimadomo D, Ubaldi N, Rienzi L, Ubaldi FM. | 2018 | Curr Opin Obstet Gynecol. | Review |
| Pretreatment With Coenzyme Q10 Improves Ovarian Response and Embryo Quality in Low-Prognosis Young Women With Decreased Ovarian Reserve: A Randomized Controlled Trial | Xu Y, Nisenblat V, Lu C, Li R, Qiao J, Zhen X, Wang S. | 2018 | Reprod Biol Endocrinol. | Randomized controlled trial |
| The Effect of Dose Adjustments in a Subsequent Cycle of Women With Suboptimal Response Following Conventional Ovarian Stimulation. | Drakopoulos P, Santos-Ribeiro S, Bosch E, Garcia-Velasco J, Blockeel C, Romito A, Tournaye H, Polyzos NP | 2018 | Front Endocrinol. (Lausanne) | Retrospective cohort study |
| Individualized controlled ovarian stimulation in expected poor-responders: an update. | Haahr T, Esteves SC, Humaidan P. | 2018 | Reprod Biol Endocrinol. | Review |
| The Novel POSEIDON Stratification of 'Low Prognosis Patients in Assisted Reproductive Technology' and Its Proposed Marker of Successful Outcome | Humaidan P, Alviggi C, Fischer R, Esteves SC. | 2016 | F1000Res. | Opinion |
| A New More Detailed Stratification of Low Responders to Ovarian Stimulation: From a Poor Ovarian Response to a Low Prognosis Concept | Poseidon Group (Patient-Oriented Strategies Encompassing IndividualizeD Oocyte Number), Alviggi C, Andersen CY, Buehler K, Conforti A, De Placido G, Esteves SC, Fischer R, Galliano D, Polyzos NP, Sunkara SK, Ubaldi FM, Humaidan P. | 2016 | Fertil Steril. | Editorial |
