# Supplementary material for: Improving Reporting of Clinical Studies Using the POSEIDON Criteria: POSORT Guidelines
Source: Front Endocrinol (Lausanne). 2021 Mar 19;12:587051. doi: 10.3389/fendo.2021.587051 (PMC8017440; doi:10.3389/fendo.2021.587051)
Supplement: Supplementary file 2 [file Table_2.docx]

**Supplementary Table 2. Information to include when reporting studies using the POSEIDON criteria***

| **Topic** | **Checklist item** | **Reported on page number (or NA)** |
| --- | --- | --- |
| **Title and abstract** | Identification as an observational study or randomized trial using the POSEIDON criteria. |  |
| **Introduction** | Explanation of rationale, specific objectives or hypotheses, and how the study may help to advance knowledge concerning the POSEIDON concept. |  |
| **Methods** |  |  |
| *Participants* | Inclusion and exclusion criteria must be clearly defined. |  |
|  | Characterize how infertility factors in participants were evaluated, describe the definitions used, and the settings where the data were collected. |  |
|  | Define which ovarian marker, AFC or AMH, or both, was used to classify the patients as per the POSEIDON criteria, and describe the methods for AFC/AMH measurements. |  |
|  | In POSEIDON groups 1 and 2 studies, previous ovarian stimulation should be characterized. |  |
| *Interventions* | Characterize the intervention (if applicable) and state the duration of the intervention noting when the treatment started and concluded. State the temporal relation of the intervention to pregnancy. |  |
| *Outcomes* | Clearly define the primary outcome. |  |
|  | When cumulative delivery rate is not the primary endpoint and embryos are transferred, reproductive outcomes (e.g., live birth delivery rate, ongoing pregnancy rate, miscarriage rate) should be reported and justified. |  |
|  | Efforts should be made to include live birth data, including gestational age, birthweight, and sex of infant. |  |
|  | Clearly define predictors, potential confounders, and effect modifiers. Describe how confounders were adjusted for. |  |
| *Data collection and analysis* | In observational studies, particularly the ones using real-world data, explain features of electronic medical records utilized, including how data quality was verified (e.g., data completeness of data, availability of data on exposure, outcomes, and covariates). |  |
|  | Describe statistical methods, including those used to control for confounders, sensitivity analyses, and how the sample size was determined. |  |
| **Results** | State the duration of infertility (including whether it is primary or secondary), relevant infertility treatment history, and cause of infertility in women and men. |  |
|  | Report the numbers of couples/patients who were screened and eligible, and describe (in observational studies) the proportion of patients fitting each POSEIDON group and those classified as non-POSEIDON. |  |
|  | Report numbers of individuals completing the follow-up and analyzed and consider the use of a flow diagram. |  |
|  | Provide unadjusted and confounder-adjusted estimates with precision (e.g., 95% confidence interval), and other analyses carried out (e.g., subgroup and sensitivity analyses). |  |
|  | Report harms^¶^ or unintended effects in each group (men, women, infants) during treatment (including both male and female partners), during pregnancy, and around birth, and in infants after birth. |  |
| **Discussion** | Discuss generalizability of study’s findings and how the results compare to other studies using the POSEIDON concept. |  |
|  | Discuss trial limitations, including but not limited to potential bias and imprecision (factors & interventions affecting endpoints should be discussed as ‘associations’ rather than ‘causation’ in observational studies). |  |

*We recommend application of these guidelines in conjunction with the CONSORT, IMPRINT, STROBE, and GRADE guidelines as appropriate (see <http://www.consort-statement.org/>; <https://strobe-statement.org/;> <https://www.graceprinciples.org/>)

^¶^Reportable harms include ovarian hyperstimulation syndrome, infection, bleeding, multiple pregnancy and maternal pregnancy complications, and harms or unintended effects on the fetus/newborn, including congenital abnormalities, and major neonatal complications as well as infant developmental delays or medical problems.

AFC: antral follicle count; AMH: anti-Müllerian hormone; NA: not applicable.
